# Iron deficiency in late pregnancy and its associations with birth outcomes in Chinese pregnant women: a retrospective cohort study

**DOI:** 10.1186/s12986-019-0360-9

**Published:** 2019-05-08

**Authors:** Xiaosong Yuan, Huiwen Hu, Ming Zhang, Wei Long, Jianbing Liu, Jian Jiang, Bin Yu

**Affiliations:** 1 Department of Prenatal Diagnosis Laboratory, Changzhou Maternity and Child Health Care Hospital Affiliated to Nanjing Medical University, 16th Ding Xiang Road, Changzhou, 213003 Jiangsu China; 2 Department of Maternity Health Care0, Changzhou Maternity and Child Health Care Hospital Affiliated to Nanjing Medical University, Changzhou, China; 3 Department of laboratory medicine, Changzhou Maternity and Child Health Care Hospital Affiliated to Nanjing Medical University, Changzhou, China; 4 Department of Obstetrics and Gynecology, Changzhou Maternity and Child Health Care Hospital Affiliated to Nanjing Medical University, Changzhou, China

**Keywords:** Iron deficiency, Ferritin, Transferrin, Low birth weight, Preterm birth, Small-for-gestational age, Large-for-gestational age, Macrosomia

## Abstract

**Background:**

Several biomarkers are used to measure iron deficiency (ID) during pregnancy, but the prevalence of ID and its association with adverse birth outcomes shows inconsistent results. The aim of this study was to examine the prevalence of ID in third trimester using multiple indicators of iron status and the relationship with birth outcomes in Chinese population.

**Methods:**

We conducted a retrospective observational cohort study of 11,581 pregnant women between 2016 and 2017 in Changzhou City, Jiangsu Province, China. We obtained the data (maternal characteristics and birth outcomes) and the concentrations of ID biomarkers from our hospitalization information system and laboratory information system, respectively. Using serum ferritin (SF), serum transferrin (ST) and their ratio as criteria of ID, we investigated associations between birth outcomes and late pregnancy ID.

**Results:**

The prevalence of ID in our study was 51.82% as defined by low SF (< 12 μg/L), 54.43% as defined by high ST (> 4 g/L) and 53.90% as defined by high ratio of ST/SF (Log 10 transform > 5.52). Maternal ST/SF ratio was associated with higher mean birth weight (97.04 g; 95% CI, 74.28, 119.81 for the highest vs. lowest quartile). Third trimester maternal ID, defined by ST/SF ratio, was associated with lower risks of preterm birth (PTB), low birth weight (LBW) and small for gestational age (SGA) infants, higher risks of macrosomia and large for gestational age (LGA) babies (for PTB: OR = 0.53, 95% CI, 0.36–0.77; for LBW: OR = 0.44, 95% CI, 0.31–0.62; for SGA: OR = 0.69, 95% CI, 0.57–0.83; for macrosomia: OR = 1.39, 95% CI, 1.13–1.70; for LGA: OR = 1.20, 95% CI, 1.04–1.39).

**Conclusions:**

ID in the third-trimester of pregnancy are frequent in Chinese women. Our findings suggest that the ratio of ST/SF measured in late pregnancy could be useful as a significant predictor of unfavorable birth outcomes.

**Electronic supplementary material:**

The online version of this article (10.1186/s12986-019-0360-9) contains supplementary material, which is available to authorized users.

## Background

Pregnant women are particularly vulnerable to iron deficiency (ID) because of increasing iron requirements of pregnancy. Globally, it is reported that the prevalence of ID varies greatly during pregnancy, ranging from 6.5 to 39.2% in United States and Canada, 28–85% in Europe, 31.4% in Korea and 19.6% in Australia, depending on the criteria used and iron supplementation [1–4]. ID is the most common cause of gestational anemia that is associated with increased risk of low birth weight (LBW) and preterm birth [5, 6]. The iron status indices in pregnant women include serum ferritin (SF), transferrin saturation, serum iron, total iron-binding capacity, red cell zinc protoporphyrin, and serum transferrin receptor (STfR). Although there is no gold standard for ID in pregnant women, it is widely accepted that pregnant women with SF levels less than 12 μg/L are classified as ID reflecting a state of iron depletion [7].

However, SF is an acute-phase protein, which is significantly increased in response to infection or inflammation. The confounding effects of inflammation may lead to misdiagnosis of ID in individuals and underestimation of the prevalence of ID in study population. To evaluate iron status in population by SF concentration, WHO currently recommends the simultaneous measurement of inflammatory markers or the exclusion of individuals with inflammation from analysis [8]. Transferrin is one of the most important protein involving in Fe homeostasis and is served as an essential biochemical marker of body iron status [9]. Compared with SF, the measurement of serum transferrin (ST) is widely available and cheap. Pregnant women with ST concentrations > 4 g/L are classified as ID [10]. Limited evidence suggests a relationship between maternal iron status and adverse birth outcomes, but this relationship seems inconsistent [11]. Furthermore, few data have been reported regarding the prevalence of ID using these multiple iron indicators from Chinese large, population-based study. The aims of this observational study were to explore the relationship between maternal iron status in the third trimester and adverse birth outcomes in Chinese pregnant women, while taking into account systemic inflammation as measured by high sensitivity C-reactive protein (hsCRP) and anemia as measured by hemoglobin.

## Methods

### Study population and laboratory measurements

This retrospective cohort study included a consecutive sample of pregnant women delivered in Changzhou Maternity and Child Health Care Hospital between April 2016 and March 2017. The ethics committee of the hospital approved the protocol of this study (No.ZD201803). We obtained the written informed consent and anonymously analyzed the data from this cohort. Based on the inclusion and exclusion criteria, we established this cohort. Inclusion criteria included: 1) pregnant women aged > 18; 2) pregnancy at 28–41 weeks of gestation; 3) integrated and clear medical records; 4) singleton pregnancy and live birth born without birth defects. Exclusion criteria were as follows: 1) multiple pregnancy; 2) missing integrated and clear medical records; 3) preexisting illnesses before getting pregnant: diabetes mellitus (type 1 or 2), chronic hypertension, thyroid diseases, chronic heart, liver and kidney diseases, immune rheumatic disease or thyroid diseases and syphilis prior to pregnancy; 4) cigarette smokers and alcohol drinkers in pregnancy. We reviewed and collected the detailed hospitalization data including maternal characteristics, history of past illness and bad hobby, pregnancy complications, labour, delivery and neonatal outcomes from our hospitalization information system. Also, laboratory information system in our hospital provided data on pregnant woman’s SF, ST and serum hsCRP values. Maternal ferritin, transferrin and hsCRP concentrations were analyzed by means of chemiluminescent immunoassay, immune turbidimetry and particle enhanced immunonephelometry using specific automated analyzers, respectively (for ferritin: UniCel DxI 800 Access, Beckman Coulter Inc., USA; for transferrin: AU5800, Beckman Coulter Inc., Japan; for hsCRP: BN II System, Siemens Diagnostics Inc., Germany). The hemoglobin was detected by hematology analyzer (Sysmex INC., Japan). Two established definitions of ID were applied: SF < 12 μg/L and ST > 4 g/L according to the previous studies [7, 10]. Based on the two definitions of ID, the ratio serum ST/SF log 10 transform was calculated and considered as another possible criterion for ID (ST/SF ratio log10 transform > 5.52).

### Definitions of the outcomes

We considered gestational diabetes mellitus (GDM), intrahepatic cholestasis of pregnancy (ICP), preeclampsia (PE) and pregnancy-induced hypertension (PIH) as major pregnancy complications; and PTB, small for gestational age (SGA), large for gestational age (LGA), LBW, macrosomia as unfavorable birth outcomes. We discriminated these abnormal outcomes from hospital data according to diagnosis by the clinician. Depending on the International Diabetic Pregnancy Association (IADPSG) criteria, 75 g oral glucose tolerance test was used to diagnose GDM at 24–28 weeks of gestation [12]. ICP always occurred in the third trimester of pregnancy, with characterization of pruritus and jaundice. The diagnosis of ICP depended on abnormal detection of liver function and increase of serum bile acid [13]. New hypertension (systolic blood pressure > 140 mmHg and/or diastolic blood pressure > 90 mmHg) with or without obvious proteinuria (> 300 mg/24 h) occurred in pregnant women with normal blood pressure after 20 weeks of pregnancy, which was diagnosed with PE or PIH, respectively [14]. PTB was defined as the birth of a newborn whose gestational age was less than 37 weeks. Neonates were divided into LBW (< 2500 g), normal birth weight (2500–4000 g) and macrosomia (> 4000 g) according to birth weight. Based on Global Reference developed by Mikolajczyk et al. [15], the average birth weight and standard deviation (SD) of 40-week-old newborns in this cohort was firstly calculated. After the mean birth weight (3513.8 g) and the coefficient of variation (11.45%) which was expressed as the percentage of SD (402.3 g) to the average birth weight at 40 weeks of gestation for the study population were input into the Microsoft Office Excel Software from the web appendix 2, the program produced multiple reference percentiles of birth weight from 24 to 41 weeks of gestation (Additional file 1: Table S1). If the birth weight fell below the 10th or exceeded the 90th percentile for gestational age, a live-birth infant was classified into SGA or LGA, respectively.

### Statistical analysis

Continuous variables with normal distribution and skewed distribution were presented as mean ± SD and median (interquartile range, IQR), respectively. Categorical variables were described as N (%). ID prevalence in our cohort was determined by these definitions of serum ferritin, transferrin and their ratio. The levels of these non-normally distributed iron biomarkers among women with or without high hsCRP (> 5 mg/L) were described by median and 25th and 75th percentiles. To compare differences between ID group and replete group, non-parametric and parametric methods were properly used to examine statistical significance. General linear analysis was applied to examine the association of serum biomarkers for ID with fetal growth (gestational age, birth length and weight). After adjusting for maternal age, BMI, gravidity, parity, hemoglobin level, Logistic regression analysis was used to explore the associations between maternal ID and pregnancy outcomes. OR and 95% CI for PTB were additionally corrected for GDM, ICP, PE, PIH, systolic and diastolic blood pressure and infant sex. These values for SGA, LGA, LBW and macrosomia were further adjusted for gestational age at delivery. Furthermore, ORs and 95% CIs for different pregnancy outcomes with quartiles of ST, SF and their ratio were evaluated by logistic regression analysis controlling for available confounders, hsCRP and hem oglobin levels. All statistical analyses were conducted using statistical software EmpowerStats (X&Y solutions Inc., USA) and R (http://www.R-project.org). A *p*-value < 0.05 was considered as statistical significance.

## Results

### Sample characteristics

A total of 11,581 mothers and their singleton newborns were included in this study after excluding 1694 women with multiple pregnancy, medical abortion, major pre-gestational diseases, congenital malformation newborns and simultaneous lack of SF and ST concentrations. The demographic characteristics of mothers and their infants were shown in Table 1. Median (IQR) maternal age and BMI at delivery were 28 (26–31) years and 26.95 (25.00–29.32), respectively. More than half (60.05%) of the pregnant women were nulliparous. The incidence of GDM, ICP, PE and PIH, which were considered as major pregnancy complications, was 8.37% (969), 6.18% (716), 3.44% (398) and 2.12% (246) respectively. Among 11,581 singleton infants, 519 (4.48%) were LBW infants and 853 (7.37%) were macrosomia. Of these babies, 1024 (8.84%) and 1793 (15.48%) were classified as SGA and LGA babies.

**Table 1:** Characteristics of mothers and newborns according to different criteria of maternal ID

	Serum ferritin (< 12 μg/L)		Serum transferrin (> 4 g/L)		Transferrin/ferritin ratio log10 transform (> 5.52)	
	Deficient (*N* = 5995)	Replete (*N* = 5574)	Deficient (*N* = 6301)	Replete (*N* = 5275)	Deficient (*N* = 6233)	Replete (*N* = 5331)
Maternal characteristics						
Maternal age at delivery (years)	28 (25–31)**	28 (26–31)	28 (25–31)**	28 (26–31)	28 (25–31)**	28 (26–32)
≤ 25 [N(%)]	1558 (25.99%)**	1168 (20.95%)	1577 (25.03%)**	1153 (21.86%)	1604 (25.73%)**	1122 (21.05%)
26–29 [N(%)]	2461 (41.05%)	2401 (43.07%)	2633 (41.79%)	2227 (42.22%)	2576 (41.33%)	2282 (42.81%)
≥ 30 [N(%)]	1976 (32.96%)**	2005 (35.97%)	2091 (33.19%)**	1895 (35.92%)	2053 (32.94%)**	1927 (36.15%)
BMI at delivery (kg/m2)	26.95 (25–29.30)	26.95 (24.98–29.38)	26.95 (24.97–29.30)	26.99 (25–29.36)	26.99 (25–29.30)	26.95 (24.97–29.36)
< 25 [N(%)]	1461 (24.65%)	1399 (25.32%)	1600 (25.63%)	1262 (24.19%)	1512 (24.53%)	1347 (25.49%)
≥25 [N(%)]	4467 (75.35%)	4126 (74.68%)	4643 (74.37%)	3955 (75.81%)	4652 (75.47%)	3937 (74.51%)
Gravidity						
< 3 [N(%)]	4138 (69.02%)**	4086 (73.30%)	4403 (69.88%)**	3825 (72.51%)	4306 (69.08%)**	3914 (73.42%)
≥3 [N(%)]	1857 (30.98%)**	1488 (26.70%)	1898 (30.12%)**	1450 (27.49%)	1927 (30.92%)**	1417 (26.58%)
Parity						
No child [N(%)]	3309 (55.20%)**	3638 (65.27%)	3629 (57.59%)**	3321 (62.96%)	3454 (55.41%)**	3489 (65.45%)
≥1 child [N(%)]	2686 (44.80%)**	1936 (34.73%)	2672 (42.41%)**	1954 (37.04%)	2779 (44.59%)**	1842 (34.55%)
Gestational age at delivery (week)	38.72 ± 1.62	38.66 ± 1.72	38.76 ± 1.54*	38.61 ± 1.81	38.73 ± 1.60	38.65 ± 1.74
Systolic BP at delivery (mmHg)	120 (110–129)**	120 (110–130)	120 (110–129)**	120 (110–130)	120 (110–129)**	120 (110–130)
Diastolic BP at delivery(mmHg)	72 (70–79)**	73 (70–80)	72 (70–80)**	73 (70–80)	72 (70–79)**	73 (70–80)
Delivery mode						
Vaginal delivery	3412 (56.91%)	2951 (52.94%)	3573 (56.71%)	3081 (58.41%)	3529 (56.62%)	3122 (58.56%)
Cesarean section	2583 (43.09%)	2334 (41.87%)	2728 (43.29%)	2194 (41.59%)	2704 (43.38%)	2209 (41.44%)
Inflammation (hsCRP > 5 mg/L)	1329 (22.17%)**	1639 (29.4%)	1465 (23.25%)**	1507 (28.57%)	1394 (22.36%)**	1572 (29.49%)
Anemia (hemoglobin < 110 g/L)	2032 (33.89%)**	396 (7.1%)	1735 (27.54%)**	692 (13.12%)	2070 (33.21%)**	356 (6.85%)
GDM	389 (6.49%)**	580 (10.41%)	539 (8.55%)	429 (8.13%)	429 (6.88%)**	539 (10.11%)
ICP	301 (5.02%)**	415 (7.45%)	448 (7.11%)**	268 (5.08%)	333 (5.34%)**	383 (7.18%)
PE	147 (2.45%)**	249 (4.47%)	190 (3.02%)*	208 (3.94%)	163 (2.62%)**	233 (4.37%)
PIH	107 (1.78%)**	139 (2.49%)	133 (2.11%)	113 (2.14%)	114 (1.83%)**	132 (2.48%)
PTB	383 (6.39%)*	411 (7.37%)	365 (5.79%)**	432 (8.19%)	389 (6.24%)**	404 (7.58%)
Newborn characteristics						
Sex						
Female	2829 (47.19%)	2623 (47.06%)	2984 (47.36%)	2473 (46.88%)	2985 (47.89%)	2465 (46.24%)
Male	3166 (52.81%)	2951 (52.94%)	3317 (52.64%)	2802 (53.12%)	3248 (52.11%)	2866 (53.76%)
Birth length (cm)	49.88 ± 1.30**	49.74 ± 1.55	49.91 ± 1.22**	49.70 ± 1.64	49.89 ± 1.28**	49.72 ± 1.58
Birth weight (g)	3390 (3110–3680)**	3320 (3030–3610)	3380 (3100–3680)**	3320 (3020–3600)	3390 (3100–3680)**	3320 (3030–3600)
< 2500	213 (3.55%)**	304 (5.45%)	209 (3.32%)**	310 (5.88%)	211 (3.39%)**	306 (5.74%)
2500–4000	5276 (88.01%)	4923 (88.32%)	5544 (87.99%)	4660 (88.34%)	5484 (87.98%)	4710 (88.35%)
> 4000	506 (8.44%)**	347 (6.23%)	548 (8.70%)**	305 (5.78%)	538 (8.63%)**	315 (5.91%)
Weight for gestational age						
SGA	428 (7.14%)**	595 (10.67%)	480 (7.62%)**	544 (10.31%)	440 (7.06%)**	583 (10.94%)
AGA	4538 (75.70%)	4217 (75.65%)	4714 (74.81%)	4045 (76.68%)	4705 (75.49%)	4045 (75.88%)
LGA	1029 (17.16%)**	762 (13.67%)	1107 (17.57%)**	686 (13.00%)	1088 (17.46%)**	703 (13.19%)

Data were presented as median (IQR), mean ± SD and N (%) for continuous variables with normal distribution, continuous variables with skewed distribution, and categorical variables, respectively. *p < 0.05, **p < 0.01, according to Mann-Whitney test for skewed-distributed continuous variables, Student’s t-test for normally-distributed continuous variables, and Chi-square test for categorical variables

Abbreviations: *IQR* interquartile range, *SD* standard deviation, *BMI* body mass index, *BP* blood pressure, *hsCRP* high sensitivity C-reactive protein, *GDM* gestational diabetes mellitus, *ICP* intrahepatic cholestasis of pregnancy, *PE* Preeclampsia, *PIH* pregnancy-induced hypertension, *PTB* pre-term birth, *SGA/AGA/LGA* small/appropriate/large for gestational age

### Maternal ID prevalence

The prevalence of ID according to SF, ST and their ratio was 51.82, 54.43 and 53.9%, respectively (Table 2). Simultaneously using all three parameters, more than one third of pregnant women (35.51%) were defined as ID. Of the 11,571 women with available hsCRP values, 25.7% (2974) had hsCRP levels > 5 mg/L, indicating inflammation. After excluding women with hsCRP > 5 mg/L, the prevalence of ID increased slightly (Table 2).

**Table 2:** Median, 25–75 percentiles and proportions of SF, ST and their ratio in late pregnancy

	N	25th percentile	Median	75th percentile	Percent deficient ^a^
All women (*N* = 11,581)					
SF (μg/L)	11,569	8.3	11.6	20.2	51.82
ST (g/L)	11,576	3.64	4.09	4.58	54.43
ST/SF ratio log10 transform	11,564	5.28	5.55	5.72	53.9
Women with hsCRP5 ≤ mg/L (*N* = 8597)					
SF (μg/L)	8591	8	11.2	19.2	54.27
ST (g/L)	8594	3.67	4.11	4.62	56.23
ST/SF ratio log10 transform	8588	5.3	5.57	5.74	56.29

^a^ Percent deficient was defined as SF < 12 μg/L, ST >  4 g/L and ST/SF ratio log10 transform > 5.52

Abbreviations: *SF* serum ferritin, *ST* serum transferrin, *hsCRP* high sensitivity C-reactive protein

### Maternal serum ferritin, transferin and fetal growth

Table 3 summarized the regression coefficient of fetal growth associated with quartiles of maternal SF, ST and their ratio. These biomarkers were respectively classified as quartile 1 (Q1), quartile 2 (Q2), quartile 3 (Q3), and quartile 4 (Q4) according to quartiles (for SF: Q1, < 8.3 μg/L; Q2, 8.3–11.5 μg/L; Q3, 11.6–20.2 μg/L; Q4, > 20.2 μg/L; for ST: Q1, < 3.64 g/L; Q2, 3.64–4.08 g/L; Q3, 4.09–4.58 g/L; Q4, > 4.58 g/L; for ST/SF ratio log10 transform: Q1, < 5.28; Q2, 5.28–5.54; Q3, 5.55–5.72; Q4, > 5.72). Maternal SF in Q2–Q4 were associated with lower birth weight relative to Q1, with estimated mean differences of − 25.56 g (95% CI, − 45.36, − 5.77), − 59.63 g (95% CI, − 80.30, − 38.97) and − 85.41 g (95% CI, − 108.23, − 62.60), respectively (p for trend < 0.01); and with lower birth length to Q1 (− 0.33 cm, 95% CI, − 0.57, − 0.09; − 0.31 cm, 95% CI, − 0.57, − 0.05; − 0.53 cm, 95% CI, − 0.81, − 0.25). ST and ST/SF ratio were positively associated with birth weight, with estimated mean increases of 76.29 g (95% CI, 56.00, 96.58) and 97.04 g (95% CI, 74.28, 119.81), respectively, for Q4 vs. Q1 (all *p* for trend < 0.01). In addition, ST showed positive association with gestational age (0.27 week, 95% CI, 0.19, 0.36) and negative association with birth length (− 0.60 cm, 95% CI, − 0.85, − 0.35), for Q4 vs. Q1 (all *p* for trend < 0.01). No statistically significant association of maternal SF with gestational age was observed.

**Table 3:** Regression coefficients [β (95% CI)] for fetal development associated with categories of SF, ST and their ratio

	Gestational age (weeks)^a^	Birth weight (g)^b^	Birth length (cm)^b^
SF (μg/L)			
< 8.3	0	0	0
8.3–11.5	0.09 (0.01, 0.18)*	−25.56 (−45.36, −5.77)*	−0.33 (− 0.57, − 0.09)**
11.6–20.2	− 0.08 (− 0.17, 0.02)	−59.63 (−80.30, −38.97)**	−0.31 (− 0.57, − 0.05)*
> 20.2	−0.09 (− 0.19, 0.01)	−85.41 (− 108.23, − 62.60)**	−0.53 (− 0.81, − 0.25)**
*P* for trend	0.0039	< 0.0001	0.0023
ST (g/L)			
< 3.64	0	0	0
3.64–4.08	0.15 (0.06, 0.24)**	33.51 (14.29, 52.73)**	−0.18 (− 0.42, 0.05)
4.09–4.58	0.25 (0.16, 0.33)**	56.15 (36.19, 76.11)**	−0.37 (− 0.62, − 0.13)**
> 4.58	0.27 (0.19, 0.36)**	76.29 (56.00, 96.58)**	−0.60 (− 0.85, − 0.35)**
*P* for trend	< 0.0001	< 0.0001	< 0.0001
ST/SF ratio log10 transform			
< 5.28	0	0	0
5.28–5.54	0.05 (− 0.04, 0.14)	27.31 (7.46, 47.16)**	0.13 (− 0.12, 0.37)
5.55–5.72	0.23 (0.15, 0.32)**	57.83 (36.69, 78.98)**	0.08 (−0.17, 0.33)
> 5.72	0.18 (0.08, 0.28)**	97.04 (74.28,119.81)**	0.22 (−0.06, 0.49)
*P* for trend	< 0.0001	< 0.0001	0.1773

^a^ Adjusted for maternal age, gravidity, parity, gestational age, BMI, systolic and diastolic BP at delivery, GDM, ICP, PE, PIH, infants sex, hsCRP (mg/L)and hemoglobin(g/L)

^b^ Additionally adjusted for gestational age

*p < 0.05, **p < 0.01

Abbreviations: *CI* confidence interval,*SF* serum ferritin, *ST* serum transferrin, *BMI* body mass index, *BP* blood pressure, *GDM* gestational diabetes mellitus, *ICP* intrahepatic cholestasis of pregnancy, *PE* Preeclampsia, *PIH* pregnancy-induced hypertension, *hsCRP* high sensitivity C-reactive protein

### ID and pregnancy outcomes

The ORs and 95% CIs for pregnancy outcomes with maternal ID were presented in Table 4. For birth outcomes, late pregnancy ID defined with these three parameters was associated with decreased risk of PTB, LBW or SGA and increased risk of macrosomia or LGA infants in all women or women with hsCRP ≤5 mg/L (for all women defined ID with SF, PTB: OR = 0.69, 95% CI, 0.58, 0.83; LBW: OR = 0.68, 95% CI, 0.49, 0.93; SGA: OR = 0.74, 95% CI, 0.63, 0.86; macrosomia: OR = 1.27, 95% CI, 1.07, 1.51;LGA: OR = 1.17, 95% CI, 1.03, 1.33; for all women defined ID with ST,PTB: OR = 0.62, 95% CI, 0.53, 0.73; LBW: OR = 0.75, 95% CI, 0.56, 1.00; SGA: OR = 0.81, 95% CI, 0.70, 0.93; macrosomia: OR = 1.46, 95% CI, 1.25, 1.71; LGA: OR = 1.36, 95% CI, 1.21, 1.52; for all women defined ID with ST/SF ratio, PTB: OR = 0.64, 95% CI, 0.54, 0.76; LBW: OR = 0.61, 95% CI, 0.44, 0.84; SGA: OR = 0.71, 95% CI, 0.61, 0.83; macrosomia: OR = 1.38, 95% CI, 1.16, 1.65; LGA: OR = 1.26, 95% CI, 1.11, 1.43; for women with hsCRP ≤5 mg/L defined ID with SF, PTB: OR = 0.71, 95% CI, 0.57, 0.88; LBW: OR = 0.58, 95% CI, 0.40, 0.84; SGA: OR = 0.71, 95% CI, 0.59, 0.85; macrosomia: OR = 1.29, 95% CI, 1.06, 1.59; LGA: OR = 1.15, 95% CI, 0.99, 1.33; for women with hsCRP ≤5 mg/L, defined ID with ST, PTB: OR = 0.63, 95% CI, 0.52, 0.76; LBW: OR = 0.75, 95% CI, 0.53, 1.06; SGA: OR = 0.77, 95% CI, 0.66, 0.91; macrosomia: OR = 1.47, 95% CI, 1.22, 1.76; LGA: OR = 1.31, 95% CI, 1.15, 1.50; for women with hsCRP ≤5 mg/L defined ID with ST/SF ratio, PTB: OR = 0.53, 95% CI, 0.36, 0.77; LBW: OR = 0.44, 95% CI, 0.31, 0.62; SGA: OR = 0.69, 95% CI, 0.57, 0.83; macrosomia: OR = 1.39, 95% CI, 1.13, 1.70; LGA: OR = 1.20, 95% CI, 1.04, 1.39). Among these associations in women with hsCRP ≤5 mg/L, for LGA with SF and for LBW with ST did not reach statistical significance (*p* = 0.06, *p* = 0.10, respectively). However, these relationships were of statistical significance when using the ratio of ST to SF as ID criterion (all *p* < 0.01 or *p* < 0.05).

**Table 4:** Multivariate analysis of pregnancy outcomes by ID criteria using SF, ST and their ratio

	SF (< 12 μg/L)	ST (> 4 g/L)	ST/SF ratio log10 transform (> 5.52)
	Adjusted OR (95%CI)	Adjusted OR (95%CI)	Adjusted OR (95%CI)
All women (*N* = 11,581)			
GDM	0.75 (0.64, 0.87)**	1.31 (1.13, 1.51)**	0.84 (0.72, 0.98)*
ICP	0.46 (0.39, 0.55)**	1.35 (1.15, 1.59)**	0.54 (0.45, 0.64)**
PE	0.49 (0.38, 0.62)**	0.80 (0.65, 1.00)	0.61 (0.49, 0.75)**
PIH	0.77 (0.57, 1.03)	1.14 (0.87, 1.49)	0.79 (0.59, 1.06)
PTB	0.69 (0.58, 0.83)**	0.62 (0.53, 0.73)**	0.64 (0.54, 0.76)**
LBW (< 2500 g)	0.68 (0.49, 0.93)*	0.75 (0.56, 1.00)*	0.61 (0.44, 0.84)**
Macrosomia (> 4000 g)	1.27 (1.07, 1.51)**	1.46 (1.25, 1.71)**	1.38 (1.16, 1.65)**
SGA	0.74 (0.63, 0.86)**	0.81 (0.70, 0.93)**	0.71 (0.61, 0.83)**
LGA	1.17 (1.03, 1.33)*	1.36 (1.21, 1.52)**	1.26 (1.11, 1.43)**
Women with hsCRP⩽5 mg/L (*N* = 8597)			
GDM	0.74 (0.62, 0.89)**	1.27 (1.08, 1.49)**	0.84 (0.71, 1.01)
ICP	0.46 (0.37, 0.56)**	1.35 (1.12, 1.63)**	0.54 (0.45, 0.66)**
PE	0.48 (0.36, 0.65)**	0.82 (0.63, 1.07)	0.60 (0.46, 0.78)**
PIH	0.74 (0.51, 1.06)	1.17 (0.84, 1.62)	0.79 (0.55, 1.13)
PTB	0.71 (0.57, 0.88)**	0.63 (0.52, 0.76)**	0.53 (0.36, 0.77)**
LBW (< 2500 g)	0.58 (0.40, 0.84)**	0.75 (0.53, 1.06)	0.44 (0.31, 0.62)**
Macrosomia (> 4000 g)	1.29 (1.06, 1.59)*	1.47 (1.22, 1.76)**	1.39 (1.13, 1.70)**
SGA	0.71 (0.59, 0.85)**	0.77 (0.66, 0.91)**	0.69 (0.57, 0.83)**
LGA	1.15 (0.99, 1.33)	1.31 (1.15, 1.50)**	1.20 (1.04, 1.39)*

Odds ratios were adjusted for maternal age, BMI, gravidity, parity, hsCRP (mg/L) and hemoglobin (g/L). Parameters of PTB were additionally corrected for GDM, ICP, PE, PIH, systolic and diastolic BP, and infant sex. The values of SGA, LGA, LBW and macrosomia were additionally corrected for gestational age at delivery.*p < 0.05, **p < 0.01

Abbreviations: *ID* iron deficiency, *SF* serum ferritin, *ST* serum transferrin, *OR* Odds ratio, *CI* confidence interval, *GDM* gestational diabetes mellitus, *ICP* intrahepatic cholestasis of pregnancy, *PE* Preeclampsia, *PIH* pregnancy-induced hypertension, *PTB* pre-term birth, *LBW* Low birth weight, *SGA/LGA* small/large for gestational age, *BMI* body mass index, *hsCRP* high sensitivity C-reactive protein, *BP* blood pressure

As regards to pregnancy complications, pregnancy ID defined with SF remained significantly associated with decreased risk of GDM, ICP and PE (GDM: OR = 0.74, 95% CI, 0.62, 0.89; ICP: OR = 0.46, 95% CI, 0.37, 0.56; PE: OR = 0.48, 95% CI, 0.36, 0.65, for women with hsCRP ≤5 mg/L). On the contrary, pregnancy ID defined with ST showed significantly associated with increased risk of GDM and ICP in all women (for GDM: OR = 1.31, 95% CI, 1.13, 1.51; for ICP: OR = 1.35, 95% CI, 1.15, 1.59) or women with hsCRP ≤5 mg/L (for GDM: OR = 1.27, 95% CI, 1.08, 1.49; for ICP: OR = 1.35, 95% CI, 1.12, 1.63). Furthermore, Table 5 showed ORs and 95% CIs for different pregnancy outcomes with quartiles of maternal SF, ST and their ratio. Increased SF was positively associated with GDM, ICP, PE, LBW, SGA (GDM, OR = 1.56, 95% CI, 1.24, 1.95; ICP, OR = 3.42, 95% CI, 2.64, 4.43; PE, OR = 3.34, 95% CI, 2.33, 4.81; LBW, OR = 2.15, 95% CI, 1.39, 3.33; SGA, OR = 1.82, 95% CI, 1.44, 2.29; for the Q4 vs. Q1, respectively; all p for trend < 0.01). Macrosomia and LGA were significantly decreased in association with the Q4 of SF (macrosomia, OR = 0.72; 95% CI, 0.56, 0.94; LGA, OR = 0.77; 95% CI, 0.64, 0.93, all *p* for trend < 0.01). Elevated ST had positive association with GDM, ICP, macrosomia and LGA (GDM, OR = 1.48, 95% CI, 1.21, 1.80; ICP, OR = 1.59, 95% CI, 1.26, 1.99; macrosomia, OR = 1.65, 95% CI, 1.31, 2.09; LGA, OR = 1.48, 95% CI, 1.26, 1.74; for the Q4 vs. Q1, respectively; all *p* for trend < 0.01). Interestingly, PTB, LBW and SGA were decreased in correlation with the Q4 of ST (PTB, OR = 0.53; 95% CI, 0.43, 0.67; LBW, OR = 0.59; 95% CI, 0.39, 0.88; SGA, OR = 0.72; 95% CI, 0.59, 0.88, *p* for trend < 0.01 or < 0.05).

**Table 5:** ORs and 95% CIs for different pregnancy outcomes with categories of SF, ST and their ratio

	GDM	ICP	PE	PIH	PTB	LBW	Macrosomia	SGA	LGA
	Adjusted OR (95%CI)	Adjusted OR (95%CI)	Adjusted OR (95%CI)	Adjusted OR (95%CI)	Adjusted OR (95%CI)	Adjusted OR (95%CI)	Adjusted OR (95%CI)	Adjusted OR (95%CI)	Adjusted OR (95%CI)
SF (μg/L)									
< 8.3	1	1	1	1	1	1	1	1	1
8.3–11.5	1.01 (0.81, 1.26)	1.52 (1.18, 1.95)**	1.40 (0.98, 1.99)	1.32 (0.87, 2.00)	0.81 (0.64, 1.02)	1.55 (1.02, 2.36)*	1.02 (0.84, 1.26)	1.33 (1.06, 1.65)*	0.97 (0.84, 1.13)
11.6–20.2	1.19 (0.95, 1.49)	2.39 (1.85, 3.08)**	1.99 (1.41, 2.82)**	1.47 (0.95, 2.27)	1.23 (0.98, 1.56)	1.91 (1.25, 2.92)**	0.85 (0.68, 1.07)	1.48 (1.18, 1.85)**	0.84 (0.72, 0.99)*
> 20.2	1.56 (1.24, 1.95)**	3.42 (2.64, 4.43)**	3.34 (2.33, 4.81)**	1.58 (1.01, 2.48)*	1.20 (0.93, 1.55)	2.15 (1.39, 3.33)**	0.72 (0.56, 0.94)*	1.82 (1.44, 2.29)**	0.77 (0.64, 0.93)**
*P* for trend	< 0.0001	< 0.0001	< 0.0001	0.112	0.036	< 0.0001	0.005	< 0.0001	0.0037
ST (g/L)									
< 3.64	1	1	1	1	1	1	1	1	1
3.64–4.08	0.99 (0.81, 1.21)	1.07 (0.85, 1.36)	0.88 (0.66, 1.17)	0.94 (0.65, 1.37)	0.78 (0.64, 0.96)*	0.81 (0.56, 1.16)	1.29 (1.01, 1.63)*	0.81 (0.68, 0.98)*	1.14 (0.96, 1.34)
4.09–4.58	1.17 (0.96, 1.43)	1.24 (0.98, 1.56)	0.74 (0.54, 1.00)*	1.02 (0.70, 1.49)	0.56 (0.45, 0.70)**	0.78 (0.53, 1.17)	1.78 (1.42, 2.24)**	0.82 (0.68, 0.99)*	1.46 (1.24, 1.71)**
> 4.58	1.48 (1.21, 1.80)**	1.59 (1.26, 1.99)**	0.78 (0.57, 1.06)	1.32 (0.91, 1.91)	0.53 (0.43, 0.67)**	0.59 (0.39, 0.88)**	1.65 (1.31, 2.09)**	0.72 (0.59, 0.88)**	1.48 (1.26, 1.74)**
*P* for trend	< 0.0001	< 0.0001	0.0795	0.1312	< 0.0001	0.0118	< 0.0001	0.0018	< 0.0001
ST/SF ratio log10 transform									
< 5.28	1	1	1	1	1	1	1	1	1
5.28–5.54	0.75 (0.62, 0.90)**	0.82 (0.67, 1.00)	0.67 (0.51, 0.88)**	1.03 (0.72, 1.45)	0.94 (0.76, 1.16)	0.84 (0.58, 1.21)	1.16 (0.92, 1.47)	0.83 (0.69, 0.99)*	1.05 (0.89, 1.24)
5.55–5.72	0.68 (0.55, 0.83)**	0.50 (0.40, 0.63)**	0.51 (0.37, 0.69)**	0.78 (0.53, 1.14)	0.57 (0.45, 0.73)**	0.69 (0.46, 1.03)	1.49 (1.17, 1.88)**	0.69 (0.56, 0.84)**	1.28 (1.08, 1.52)**
> 5.72	0.75 (0.60, 0.94)*	0.41 (0.32, 0.53)**	0.33 (0.22, 0.48)**	0.85 (0.55, 1.32)	0.67 (0.52, 0.86)**	0.36 (0.23, 0.57)**	1.44 (1.12, 1.86)**	0.51 (0.41, 0.65)**	1.39 (1.16, 1.66)**
*P* for trend	< 0.0001	< 0.0001	< 0.0001	0.2983	< 0.0001	0.0001	0.0011	< 0.0001	< 0.0001

Odds ratios were adjusted for maternal age, BMI, gravidity, parity, hsCRP (mg/L) and hemoglobin (g/L). Parameters of PTB were additionally corrected for GDM, ICP, PE, PIH, systolic and diastolic BP, and infant sex. The values of SGA, LGA, LBW and macrosomia were additionally corrected for gestational age at delivery.*p < 0.05, **p < 0.01

Abbreviations: *ID* iron deficiency, *SF* serum ferritin, *ST* serum transferrin, *OR* Odds ratio*. CI* confidence interval, *GDM* gestational diabetes mellitus, *ICP* intrahepatic cholestasis of pregnancy, *PE* Preeclampsia, *PIH* pregnancy-induced hypertension, *PTB* pre-term birth, *LBW* Low birth weight, *SGA/LGA* small/large for gestational age, *BMI* body mass index, *hsCRP* high sensitivity C-reactive protein, *BP* blood pressure

## Discussion

To our knowledge, this is the largest report to investigate the prevalence of ID and adverse outcomes in Chinese pregnant women. Our study shows that more than half of Chinese pregnant women are ID based on SF, ST and their ratio during late pregnancy. ID in the third trimester is associated with increased risk for macrosomia and LGA infants, and decreased risk of PTB, LBW and SGA babies. Up till to now, there have no published data on the association between maternal ST concentrations and subsequent birth outcomes. In this retrospective observational study, we also examined the association of the quartiles of ST and ST/SF ratio with birth outcomes. Maternal ST concentrations and ST/SF ratio are positively associated with the risk of macrosomia and LGA babies and negatively associated with the risk for PTB, LBW and SGA newborns.

Compared with anemia diagnosed by hemoglobin, there is fewer reports on the associations of maternal iron status classified by SF with adverse birth outcomes. Furthermore, available findings show inconsistent results. In early pregnancy, low SF concentrations (< 15 μg/L) were associated with increased risk of SGA in one study, and a high level of SF (>75th percentile) was only correlated with an elevated risk of PTB in another report, however, no significant associations of ID (SF < 12 μg/L) with birth outcomes were observed in the third study [4, 16, 17]. During the second trimester of pregnancy, both high SF concentration and ID (SF < 12 μg/L) were contradictorily reported to be correlated to LBW and PTB in different studies, but no correlations were found in one study while evaluating these outcomes [18–21]. Overall, in late pregnancy, only high SF concentrations were associated with increased risks of LBW and/or PTB in previous studies [18, 21, 22]. Although our observational subjects collected blood samples before delivery, which was later than previous studies, similar associations were still found in 11,581 Chinese pregnant women. We confirmed that a high SF is not only associated with high risks of SGA and LBW, but also with low risks of LGA and macrosomia. However, the association of high SF with PTB did not obtain statistical significance. The underlying mechanism that high iron status classified by a high SF in late pregnancy had unfavorable effects on birth outcomes remains unclear. High iron status in late pregnancy may affect birth outcomes in three ways: increasing blood viscosity and reducing placental blood flow, leading to oxidative stress, suppressing the systemic response to inflammation or infection [23–26]. In addition, these findings probably reflect the presence of inflammation or infection in the third trimester of pregnancy, thereby increasing SF rather than high iron status.

Furthermore, the present study suggested maternal ID defined by SF < 12 μg/L in late pregnancy was significantly associated with decreased risk for PTB, LBW and SGA neonates. As we know, it is the larger observational cohort study correlated reduced SF concentration (< 12 μg/L) in the third trimester with low risk of these adverse birth outcomes. Our findings were inconsistent with previous reports the relationship between ID and these pregnancy outcomes with some reporting no a relationship between ID and birth outcomes [4, 18, 22], while others observed a relationship with high risk of birth outcomes [16, 20, 27]. The inconsistent result of relationship may be explained by studies carried out in different populations and/or trimester of pregnancy, study design (case control or cohort), size of sample and lack of controlling for important confounding variables such as low grade inflammation in statistical analyse. For example, the three studies with findings contrary to us were conducted in the first and second trimester of pregnancy. It has also been known that the relationship between maternal hemoglobin level and birth outcomes differ by trimester [11]. Scanlon et al. investigated the risk of SGA among 173,371 pregnant women from the United States based on maternal hemoglobin level [28]. They observed that pregnant women with a high hemoglobin level in the first and second trimester had an increased risk of SGA; this was not obvious for the hemoglobin level in late pregnancy, but those with a hemoglobin concentration below the reference range in the third trimester had a decreased risk of SGA. Different correlations by trimester were also observed in a Chinese study of 164,667 women [29, 30]; high risk of PTB were found among women with a low hemoglobin level in the first trimester, but there was little correlation with the hemoglobin level in the second trimester, and the relationship was reversed in late pregnancy (low risk of PTB and low hemoglobin). In addition, Wang et al. demonstrated that a U-shaped relationship between hemoglobin level and risk of LBW differs by trimesters [31]. We hypothesized that SF may regulate the concentration of hemoglobin and affect unfavorable birth outcomes probably differed by trimesters. In order to confirm this, a prospective longitudinal study with large sample size focusing on the association of SF with unfavorable outcomes is urgently necessary.

ID is considered to be the most common nutritional deficit in the world. The prevalence of ID in developing countries is far greater than that in developed countries [32]. Insufficient intake is the most common cause in the former, while other important diseases may be related to the cases of the latter. There was only two studies involving in the prevalence of ID among pregnant women in China with which to compare the present results [33, 34]. Ma et al. recruited 734 clinically normal pregnant women in the third trimester for micronutrient and hematologic evaluation. ID (SF < 12 μg/L) in 734 cases was 44.7%, which was lower than 51.82% in our study. One limitation of their study was the lack of data on inflammation markers. The confounding effects of inflammation may contribute to a reduction in the prevalence of ID when the SF level is used as the criterion of ID. Our findings showed that the ID prevalence increased from 51.82 to 54.27% after excluding those cases with possible inflammation (hsCRP > 5 mg/L), which confirmed this situation. Moreover, China is the largest developing country with a wide diversity of economy, living environments and dietary habits. To some extent, the prevalence of ID varies with the utilization of different study population and sample sizes. Liao et al. investigated the concentration of SF in 3591 pregnant women from 15 provinces in China and demonstrated late pregnancy ID was 51.6%, which was very close to our finding [34].

In terms of birth outcomes, previous study by Khambalia et al. found that ID defined by total body iron (TBI), but not SF and STfR was associated with increased risk of LGA infants [4]. Our findings also suggested that ID, defined using ST and ST to SF ratio, but not SF, was correlated with elevated risk of LGA. In our study, we did not calculate TBI from participants without STfR concentrations. Although SF may be the most effective detection of ID in the absence of inflammation, many laboratories continue to provide ST measurements as an alternative to SF in some cases because of lower detection cost. In a direct comparison by Hawkins et al., ST test is superior to iron and saturation measurement in predicting iron deficiency [35]. In our study, we conducted combinational measurements of ST and SF, and calculated their ratios of log 10 transformation. We found that ID defined using the ratio of ST/SF, rather than ST or SF, is significantly associated with major birth outcomes (PTB, LBW, SGA, LGA, macrosomia) in these subjects (hsCRP ≤5 mg/L) or all the study population. Our findings suggest that ST/SF ratio could be used as a significant predictor of adverse birth outcomes.

Several strengths of the present cohort study included as follows: 1) sufficient sample size of pregnant women with different levels of ferritin and transferrin or major birth outcomes, 2) some potential confounders were firstly excluded before analysis including subjects with most pre-existing diseases or fetal congenital malformation, and known confounding factors such as maternal age and BMI or hsCRP and hemoglobin level were also corrected by logistic regression analysis, 3) the levels of ID biomarkers as well as hsCRP, hemoglobin and the birth outcomes were prospectively documented in laboratory and hospitalization database, respectively, 4) all the tests were carried out in the same laboratory using same machines with same settings, 5) our hospital network included the rural and urban populations reflecting the general population rather than the referral center for high-risk women. However, the current study still has some limitations. Because of the use of a retrospective database, we were unable to investigate the association of maternal ID with risk of adverse pregnancy outcomes in the first and second trimester. The third trimester of pregnancy is the most important period for fetal growth, however, it is believed that the majority of late pregnancy disorders originate from earlier pregnancy [36]. It is uncertain which trimester of pregnancy is most relevant, longitudinal investigation of serum ID biomarkers during different periods of pregnancy would be helpful to explain this question in future studies. Also, the database in our hospital did not provide the information on maternal detailed characteristics including pre-pregnancy BMI, weight gain during pregnancy, socio-economic level (poverty and education) and Fe supplementation (iron rich food). Lack of controlling for these variables might contribute to possible statistical bias or overestimation of the risk of unfavorable birth outcomes. To further investigate the development of ID before, during and after pregnancy and to evaluate its relationship with pregnancy outcomes, it is necessary to conduct more prospective multicenter studies with larger population.

## Conclusion

More than half of Chinese women have experienced late pregnancy ID, which gives the importance of routine screening for anemia in pregnant women and iron research for suspected ID women. The correlation between ID defined by ST/SF ratio and risks of adverse birth outcomes requires further investigation in other study populations. Our findings and those reported in other studies suggest that if increased SF levels are true reflection of iron excess in mothers, and are associated with adverse birth outcomes, the rationality of routine iron supplementation for all pregnant women should be reexamined.

## Additional file

Additional file 1: **Table S1.** Percentiles of birth weight for a population with the mean birth weight at 40 weeks of gestation of 3513.8 g in China. (DOCX 27 kb)

## Abbreviations

BMI: Body mass index
CI: Confidence interval
GDM: Gestational diabetes mellitus
hsCRP: High sensitivity C-reactive protein
ICP: Intrahepatic cholestasis of pregnancy
ID: Iron deficiency
IQR: Interquartile range
LBW: Low birth weight
LGA: Large for gestational age
OR: Odds ratio
PE: Preeclampsia
PIH: Pregnancy-induced hypertension
PTB: Preterm birth
SD: Standard deviation
SF: Serum ferritin
SGA: Small for gestational age
ST: Serum transferrin
STfR: Serum transferrin receptor
