# Supplementary material for: Iron deficiency in late pregnancy and its associations with birth outcomes in Chinese pregnant women: a retrospective cohort study
Source: Nutr Metab (Lond). 2019 May 8;16:30. doi: 10.1186/s12986-019-0360-9 (PMC6505078; doi:10.1186/s12986-019-0360-9)
Supplement: Supplementary file 1 — Table S1. Percentiles of birth weight for a population with the mean birth weight at 40 weeks of gestation of 3513.8 g in China. (DOCX 27 kb) [file 12986_2019_360_MOESM1_ESM.docx]

| **Table 1 Characteristics of mothers and newborns according to different cut off values of serum ferritin for diagnosis of maternal ID** | | | | |
| --- | --- | --- | --- | --- |
|  | Serum ferritin ( <12 μg/L) | | Serum ferritin ( ≤20 μg/L) | |
|  | Deficient (N=5995) | Replete (N=5574) | Deficient (N=8647) | Replete (N=2922) |
| Maternal characteristics |  |  |  |  |
| Maternal age at delivery (years) | 28 (25–31)** | 28 (26–31) | 28 (26–31)** | 28 (26–32) |
| ≤ 25 [N(%)] | 1558 (25.99%)** | 1168 (20.95%) | 2154 (24.91%)** | 572 (19.58%) |
| 26–29 [N(%)] | 2461 (41.05%) | 2401 (43.07%) | 3610 (41.75%) | 1252 (42.85%) |
| ≥ 30 [N(%)] | 1976 (32.96%)** | 2005 (35.97%) | 2883 (33.34%)** | 1098 (37.58%) |
| BMI at delivery (kg/m2) | 26.95 (25–29.30) | 26.95 (24.98–29.38) | 26.95 (24.98–29.30) | 26.99 (25–29.40) |
| <25 [N(%)] | 1461 (24.65%) | 1399 (25.32%) | 2145 (25.06%) | 715 (24.71%) |
| ≥25 [N(%)] | 4467 (75.35%) | 4126 (74.68%) | 6414 (74.94%) | 2179 (75.29%) |
| Gravidity |  |  |  |  |
| <3 [N(%)] | 4138 (69.02%)** | 4086 (73.30%) | 6031 (69.75%)** | 2193 (75.05%) |
| ≥3 [N(%)] | 1857 (30.98%)** | 1488 (26.70%) | 2616 (30.25%)** | 729 (24.95%) |
| Parity |  |  |  |  |
| No child [N(%)] | 3309 (55.20%)** | 3638 (65.27%) | 4971 (57.49%)** | 1976 (67.62%) |
| ≥1 child [N(%)] | 2686 (44.80%)** | 1936 (34.73%) | 3676 (42.51%)** | 946 (32.38%) |
| Gestational age at delivery (week) | 38.72 ± 1.62 | 38.66 ± 1.72 | 38.70 ± 1.67 | 38.66 ± 1.67 |
| Systolic BP at delivery (mmHg) | 120 (110–129)** | 120 (110–130) | 120 (110–129)** | 120 (110–130) |
| Diastolic BP at delivery(mmHg) | 72 (70–79)** | 73 (70–80) | 72 (70–80)** | 74 (70–80) |
| Delivery mode |  |  |  |  |
| Vaginal delivery | 3412 (56.91%) | 2951 (52.94%) | 4947 (57.21%) | 1705 (58.35%) |
| Cesarean section | 2583 (43.09%) | 2334 (41.87%) | 3700 (42.79%) | 1217 (41.65%) |
| Inflammation (hsCRP >5 mg/L) | 1329 (22.17%)** | 1639 (29.4%) | 2096 (24.26%)** | 872 (29.85%) |
| Anemia ( hemoglobin < 110 g/L) | 2032 (33.89%)** | 396 ( 7.1%) | 2275 (26.31%)** | 153 (5.24%) |
| GDM | 389 ( 6.49%)** | 580 (10.41%) | 621 ( 7.89%)** | 348 ( 13.56%) |
| ICP | 301 ( 5.02%)** | 415 ( 7.45%) | 481 ( 6.22%)** | 235 ( 9.58%) |
| PE | 147 ( 2.45%)** | 249 ( 4.47%) | 244 ( 3.26%)** | 152 ( 6.41%) |
| PIH | 107 ( 1.78%)** | 139 ( 2.49%) | 170 ( 2.29%)** | 76 ( 3.31%) |
| PTB | 383 ( 6.39%)* | 411 ( 7.37%) | 580 ( 6.71) | 214 ( 7.32%) |
| Newborn characteristics |  |  |  |  |
| Sex |  |  |  |  |
| Female | 2829 (47.19%) | 2623 (47.06%) | 4106 (47.48%) | 1346 (46.06%) |
| Male | 3166 (52.81%) | 2951 (52.94%) | 4541 (52.52%) | 1576 (53.94%) |
| Birth length (cm) | 49.88 ± 1.30** | 49.74 ± 1.55 | 49.84 ± 1.37** | 49.72 ± 1.58 |
| Birth weight (g) | 3390 (3110–3680)** | 3320 (3030–3610) | 3370 (3090–3660)** | 3300 (3020–3600) |
| <2500 | 213 ( 3.55%)** | 304 ( 5.45%) | 350 ( 4.05%)** | 167 ( 5.72%) |
| 2500–4000 | 5276 (88.01%) | 4923 (88.32%) | 7608 (87.98%) | 2591 (88.67%) |
| >4000 | 506 ( 8.44%)** | 347 ( 6.23%) | 689 ( 7.97%)** | 164 ( 5.61%) |
| Weight for gestational age |  |  |  |  |
| SGA | 428 ( 7.14%)** | 595 (10.67%) | 676 ( 7.82%)** | 347 (11.88%) |
| AGA | 4538 (75.70%) | 4217 (75.65%) | 6556 (75.82%) | 2199 (75.26%) |
| LGA | 1029 (17.16%)** | 762 (13.67%) | 1415 (16.36%)** | 376 (12.87%) |
| Data were presented as median (IQR), mean ±SD and N (%) for continuous variables with normal distribution, continuous variables with skewed distribution, and categorical variables, respectively.  *p < 0.05, **p < 0.01, according to Mann-Whitney test for skewed-distributed continuous variables, Student’s t-test for normally-distributed continuous variables, and Chi-square test for categorical variables.  Abbreviations: IQR interquartile range, SD standard deviation, BMI body mass index, BP blood pressure, hsCRP high sensitivity C-reactive protein, GDM gestational diabetes mellitus, ICP intrahepatic cholestasis of pregnancy, PE Preeclampsia, PIH pregnancy-induced hypertension, PTB pre-term birth, SGA/AGA/LGA small/appropriate/large for gestational age | | | | |

| **Table 2** Multivariate analysis of pregnancy outcomes by ID criteria using SF <12 μg/L and SF ≤20 μg/L | | |
| --- | --- | --- |
|  | SF ( <12 μg/L) | SF ( ≤20 μg/L) |
|  | Adjusted OR (95%CI) | Adjusted OR (95%CI) |
| All women (N= 11581) |  |  |
| GDM | 0.75 (0.64, 0.87)** | 0.72 (0.61, 0.84)** |
| ICP | 0.46 (0.39, 0.55)** | 0.51 (0.42, 0.61)** |
| PE | 0.49 (0.38, 0.62)** | 0.48 (0.38, 0.62)** |
| PIH | 0.77 (0.57, 1.03) | 0.85 (0.63, 1.15) |
| PTB | 0.69 (0.58, 0.83)** | 0.82 (0.67, 0.99)* |
| LBW (<2500g) | 0.68 (0.49, 0.93)* | 0.75 (0.54, 1.04) |
| Macrosomia (>4000g) | 1.27 (1.07, 1.51)** | 1.30 (1.06, 1.60)* |
| SGA | 0.74 (0.63, 0.86)** | 0.73 (0.62, 0.86)** |
| LGA | 1.17 (1.03, 1.33)* | 1.18 (1.02, 1.36)* |
| Women with hsCRP ⩽5 mg/L (N = 8597) |  | |
|  |  |  |
| GDM | 0.74 (0.62, 0.89)** | 0.68 (0.57, 0.82)** |
| ICP | 0.46 (0.37, 0.56)** | 0.49 (0.40, 0.61)** |
| PE | 0.48 (0.36, 0.65)** | 0.48 (0.35, 0.65)** |
| PIH | 0.74 (0.51, 1.06) | 0.84 (0.57, 1.24) |
| PTB | 0.71 (0.57, 0.88)** | 0.79 (0.62, 1.01) |
| LBW (<2500g) | 0.58 (0.40, 0.84)** | 0.57 (0.38, 0.85)** |
| Macrosomia (>4000g) | 1.29 (1.06, 1.59)* | 1.34 (1.05, 1.71)* |
| SGA | 0.71 (0.59, 0.85)** | 0.71 (0.59, 0.86)** |
| LGA | 1.15 (0.99, 1.33) | 1.18 (1.00, 1.40) |
| Odds ratios were adjusted for maternal age, BMI, gravidity, parity, hsCRP (mg/L) and hemoglobin (g/L). Parameters of PTB were additionally corrected for GDM, ICP, PE, PIH, systolic and diastolic BP, and infant sex. The values of SGA, LGA, LBW and macrosomia were additionally corrected for gestational age at delivery.**p*< 0.05, ***p*< 0.01.  Abbreviations: ID iron deficiency, SF serum ferritin, ST serum transferrin, OR Odds ratio  *CI* confidence interval, *GDM* gestational diabetes mellitus, *ICP* intrahepatic cholestasis of pregnancy, *PE* Preeclampsia, *PIH* pregnancy-induced hypertension, *PTB* pre-term birth, LBW Low birth weight, *SGA/LGA* small/large for gestational age, *BMI* body mass index, *hsCRP* high sensitivity C-reactive protein, *BP* blood pressure | | |
